# Response: Commentary: Reducing Viability Bias in Analysis of Gut Microbiota in Preterm Infants at Risk of NEC and Sepsis

**DOI:** 10.3389/fcimb.2018.00374

**Published:** 2018-10-24

**Authors:** Gregory R. Young, Darren L. Smith, Nicholas D. Embleton, Janet Elizabeth Berrington, Edward C. Schwalbe, Stephen Paul Cummings, Christopher J. van der Gast, Clare Lanyon

**Affiliations:** ^1^Faculty of Health and Life Sciences, University of Northumbria, Newcastle upon Tyne, United Kingdom; ^2^Newcastle Neonatal Service, Newcastle upon Tyne Hospitals NHS Foundation Trust, Newcastle upon Tyne, United Kingdom; ^3^School of Science and Engineering, Teesside University, Middlesbrough, United Kingdom; ^4^School of Healthcare Science, Manchester Metropolitan University, Manchester, United Kingdom

**Keywords:** PMA, viability analyses, microbiota, methods—analytic, bias, clinical samples

We would like to thank Agustí and Codony (2018), for their interest in our recent manuscript (Young et al., 2017), and valuable comments. We agree that non-viable cell exclusion is an important consideration, worth making when conducting analyses of microbial communities via targeted DNA sequencing and amplification approaches. The technique is especially pertinent in environments where large volumes of non-viable bacteria are expected, such as in preterm infant stool, where multiple clinical interventions manifest deliberate bacterial killing.

Whilst we agree with the general principles with regards to sample collection and handling described by Agustí and Codony (2018) we maintain that these are not always possible in real-life, clinical patient samples. For example, as patient care is the primary concern in this cohort, sample collection is convenience based rather than experimentally dictated. This requires samples to be spontaneously collected and stored prior to processing. We concede this increases the likelihood of loss of anaerobic bacterial viability. We would, however point out that the study by Brusa et al. (1989), highlighted in the commentary defines viability purely as culturability. Studies (Contreras et al., 2011; Nocker et al., 2011) have reported loss of culturability occurs at lower stress levels than loss of membrane integrity. Thus, suggesting greater cellular stress may be required to deplete DNA signals from non-viable cells, as determined during PMA-based viability assays. Moreover, we highlight in our manuscript that PMA is a conservative parameter for loss of viability. We also propose that, in the specific cohort investigated, conservative non-viable determination is preferable to over-exaggerated determination or a complete absence of it. This is especially true when non-treated samples can be analyzed in parallel.

DNA retention on microtube surfaces and subsequent inclusion in the viable communities may well be an issue in PMA-based viability determination. In our study however, samples were not heat-killed at any point. This is in contrast to the study by Agustí et al. (2017), in which DNA binding to microtube walls may have occurred prior to PMA-treatment. Furthermore, the methods described by ourselves outline transfer between sterile glass pots during initial sample collection and several microtubes and well plates from PMA-addition to incubation and photo-activation and, finally DNA isolation. We propose that these several steps are sufficient to reduce the impact of DNA retained on microtube surfaces on the assigned live DNA fraction.

In addition to the valuable comments made by Agustí and Codony (2018), where the target sequence is < 300 bp we recommend combination of a nested-PCR approach, comprising initial long fragment pre-amplification, followed by subsequent target amplicon sequencing/quantification, first described by Luo et al. (2010). This increases the likelihood of encountering intercalated PMA during the DNA amplification process.

The use of PMA as a viability-dye is extremely useful in microbial community analysis. As a greater volume of literature becomes available regarding it's use, the technique will no doubt refine. From our experience with the technique, we propose several important considerations to be made when planning any study in which PMA-based non-viable cell exclusion is employed:

- Is the sample likely to contain substantial volumes of DNA originating from non-viable bacteria?
- Are the collection and storage methods likely to impact community viability?
- What is the composition of the sample? (Homogenisation may be required)
- What is the optimal concentration of PMA required to ensure non-viable cell DNA quenching?
- Is there potential for extra-cellular DNA carry-over in the methods? (specifically from DNA retained on plastics surfaces)
- What is my light-source for photoactivation (464 nm is excitation maxima of PMA therefore substantial emission at this wavelength is required)
- Can I employ a nested-PCR approach to maximize intercalated PMA impact?
- Can I afford to sequence the non-treated sample also? (Particularly useful in clinical environments to ID bacterial communities that may have been viable recently)
- Can I afford to supplement this technique with another to confirm presence/viability? (e.g., FISH, flow cytometry, selective culture)

We would further direct anyone interested to a comprehensive review of the technique by Fittipaldi et al. (2012).

## Author contributions

GY wrote the response. All authors proofread and approved this commentary response.

### Conflict of interest statement

The authors declare that the research was conducted in the absence of any commercial or financial relationships that could be construed as a potential conflict of interest.
